# Joubert syndrome-derived induced pluripotent stem cells show altered neuronal differentiation in vitro

**DOI:** 10.1007/s00441-024-03876-9

**Published:** 2024-03-19

**Authors:** Roberta De Mori, Silvia Tardivo, Lidia Pollara, Silvia Clara Giliani, Eltahir Ali, Lucio Giordano, Vincenzo Leuzzi, Rita Fischetto, Blanca Gener, Santo Diprima, Marco J. Morelli, Maria Cristina Monti, Virginie Sottile, Enza Maria Valente

**Affiliations:** 1 Induced Pluripotent Stem Cells Unit, IRCCS Santa Lucia Foundation, Rome, Italy; 2 Neurogenetics Lab, IRCCS Santa Lucia Foundation, Rome, Italy; 3 Neurogenetics Research Unit, IRCCS Mondino Foundation, Pavia, Italy; 4 Department of Molecular and Translational Medicine, University of Brescia, Brescia, Italy; 5 Paediatric Neurology and Psychiatry Unit, Spedali Civili Children’s Hospital, University of Brescia, Brescia, Italy; 6 Unit of Child Neurology and Psychiatry, Department of Human Neuroscience, University of Rome La Sapienza, Rome, Italy; 7 Clinical Genetics Unit, Department of Pediatric Medicine, XXIII Children’s Hospital, Bari, Giovanni Italy; 8 Department of Genetics, Cruces University Hospital, BioBizkaia Health Research Institute, 48903 Barakaldo Cruces PlazaBizkaia, Spain; 9 IRCCS San Raffaele Scientific Institute, Milan, Italy; 10 Unit of Biostatistics and Clinical Epidemiology, Department of Public Health, Experimental and Forensic Medicine, University of Pavia, Pavia, Italy; 11 Department of Molecular Medicine, University of Pavia, Pavia, Italy

**Keywords:** Joubert syndrome, iPSCs, Cerebellum, Primary cilium, Ciliopathies

## Abstract

Joubert syndrome (JS) is a recessively inherited congenital ataxia characterized by hypotonia, psychomotor delay, abnormal ocular movements, intellectual disability, and a peculiar cerebellar and brainstem malformation, the “molar tooth sign.” Over 40 causative genes have been reported, all encoding for proteins implicated in the structure or functioning of the primary cilium, a subcellular organelle widely present in embryonic and adult tissues. In this paper, we developed an *in vitro* neuronal differentiation model using patient-derived induced pluripotent stem cells (iPSCs), to evaluate possible neurodevelopmental defects in JS. To this end, iPSCs from four JS patients harboring mutations in distinct JS genes (*AHI1, CPLANE1*, *TMEM67*, and *CC2D2A*) were differentiated alongside healthy control cells to obtain mid-hindbrain precursors and cerebellar granule cells. Differentiation was monitored over 31 days through the detection of lineage-specific marker expression by qRT-PCR, immunofluorescence, and transcriptomics analysis. All JS patient-derived iPSCs, regardless of the mutant gene, showed a similar impairment to differentiate into mid-hindbrain and cerebellar granule cells when compared to healthy controls. In addition, analysis of primary cilium count and morphology showed notable ciliary defects in all differentiating JS patient-derived iPSCs compared to controls. These results confirm that patient-derived iPSCs are an accessible and relevant *in vitro* model to analyze cellular phenotypes connected to the presence of JS gene mutations in a neuronal context.

**Supplementary Information:**

The online version contains supplementary material available at 10.1007/s00441-024-03876-9.

## Introduction

Ciliopathies are an expanding group of disorders due to mutations in genes involved in the formation and function of the primary cilium, a subcellular organelle implicated in key biological processes both during embryonic and adult life. Ciliopathies affect several organs such as the central nervous system (CNS), retina, kidneys, liver, and skeleton, reflecting the multiple roles of primary cilia in different tissues (Mitchison and Valente 2017). They are both genetically heterogeneous and pleiotropic, as in the case of Joubert syndrome (JS, MIM213300), a ciliopathy characterized by a unique cerebellar and brainstem malformation termed the “molar tooth sign” (MTS). This consists of cerebellar vermis dysplasia, a deepened interpeduncular fossa, and thickened, elongated, and poorly oriented superior cerebellar peduncles (SCPs), which often fail to decussate (Romani et al. 2013). Clinically, neurological manifestations of JS comprise developmental delay, oculomotor apraxia, hypotonia, ataxia, intellectual deficit, and breathing dysregulation in the neonatal age. Other manifestations may involve eye (Leber congenital amaurosis, retinal dystrophy), kidney (nephronophthisis, polycystic kidney disease), skeleton (mainly polydactyly), liver (congenital fibrosis), and oral-facial and laterality defects, defining heterogeneous phenotypic subgroups sharing the MTS (Mitchison and Valente 2017).

More than 40 genes have been identified to date, overall accounting for about two-thirds of the cases. Despite this extreme genetic heterogeneity, five JS major genes (*TMEM67, AHI1, CPLANE1, CEP290*, and *CC2D2A*) were found to account for about half of genetically characterized cases (Bachmann-Gagescu et al. 2015; Mitchison and Valente 2017). Interestingly, while dysfunction of all these genes results in the same specific brain malformation (the MTS), they encode for proteins involved in different aspects of cilia biology: (i) TMEM67 is part of the Tectonic Complex within the transition zone (TZ), a specialized region of the cilium acting as ciliary gate and regulating both ciliogenesis and ciliary membrane composition (Garcia-Gonzalo et al. 2011); moreover, TMEM67 has been implicated in non-canonical Wnt signaling (Abdelhamed et al. 2015; Lee et al. 2017); (ii) AHI1 is located at the basal body and is known to stabilize the canonical Wnt pathway effector beta-catenin (Lancaster et al. 2011); (iii) CPLANE1, part of the CPLANE complex, resides at the basal body and has recently been related to Kif7 and the Shh pathway (Asadollahi et al. 2018); (iv) CEP290 localizes at the TZ where it plays an important regulatory role, preventing inappropriate entry of membrane-associated proteins into cilia and regulating the assembly pathway of the various protein complexes to build a functional TZ (Li et al. 2016); (v) finally, CC2D2A is a TZ protein involved in the organization of the vesicle fusion machinery at the periciliary membrane (Ojeda Naharros et al. 2017). So far, the role of the primary cilium in the developing cerebellum was only partially elucidated. It still remains unclear how malfunction of the proteins which impair cilia biology at different levels may eventually result in such a consistent neurodevelopmental defect as the MTS.

The development of induced pluripotent stem cells (iPSCs) from somatic cells represents a simple and elegant model to study human diseases, providing the means to maintain the specific patient’s genotype *in vitro*. iPSCs possess a cilium (Barabino et al. 2020) and can differentiate into almost every cell type, representing a powerful model to study the effect of selected mutations on the differentiation process leading to specific phenotypes. Here, we established iPSCs from JS patients carrying mutations in four different major genes (*AHI1*, *TMEM67*, *CPLANE1*, and *CC2D2A*) and characterized their ciliary and differentiation properties compared to iPSCs derived from healthy controls.

## Materials and methods

### Generation of iPSCs from control and JS patients

**Fig. 1:**
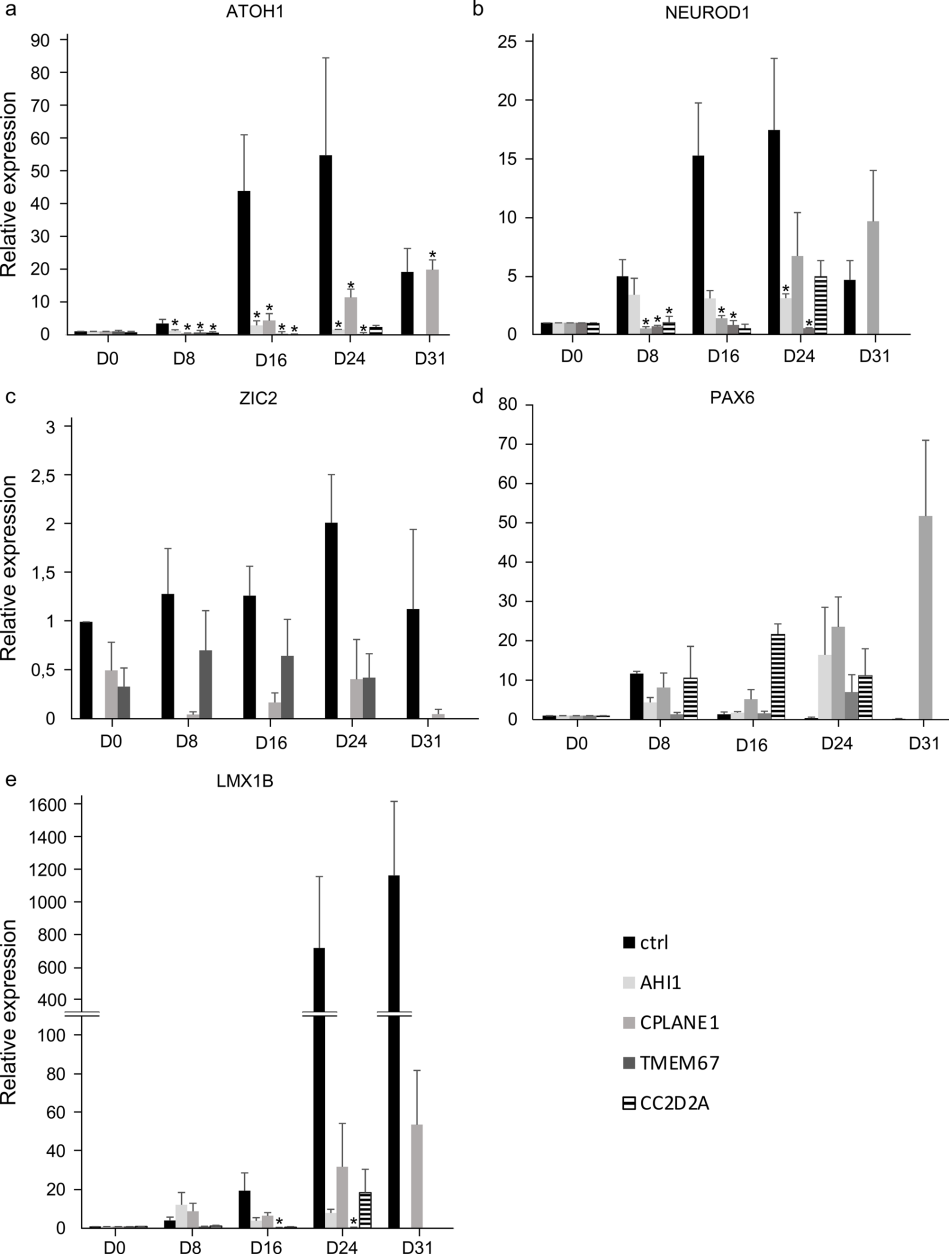
Changes in gene expression in control and JS-derived iPSCs over the 31-day differentiation treatment. Relative fold change in the expression of *ATOH1* (**a**), *NEUROD1* (**b**), *ZIC2* (**c**), *PAX6* (**d**), and *LMX1B* (**e**) measured in each JS line normalized to D0 and then compared to the mean of CT-iPSC values. Only the *CPLANE1* JS-iPSC line survived up to the day 31 time-point. *n* = 3 biological replicates (with 2 technical replicates for each); * is shown when the comparison, at each time point, of each JS-iPSCs vs CT-iPSCs (ctrl) is statistically significant (*p* < 0.05) using Wilcoxon-Mann–Whitney tests

**Fig. 2:**
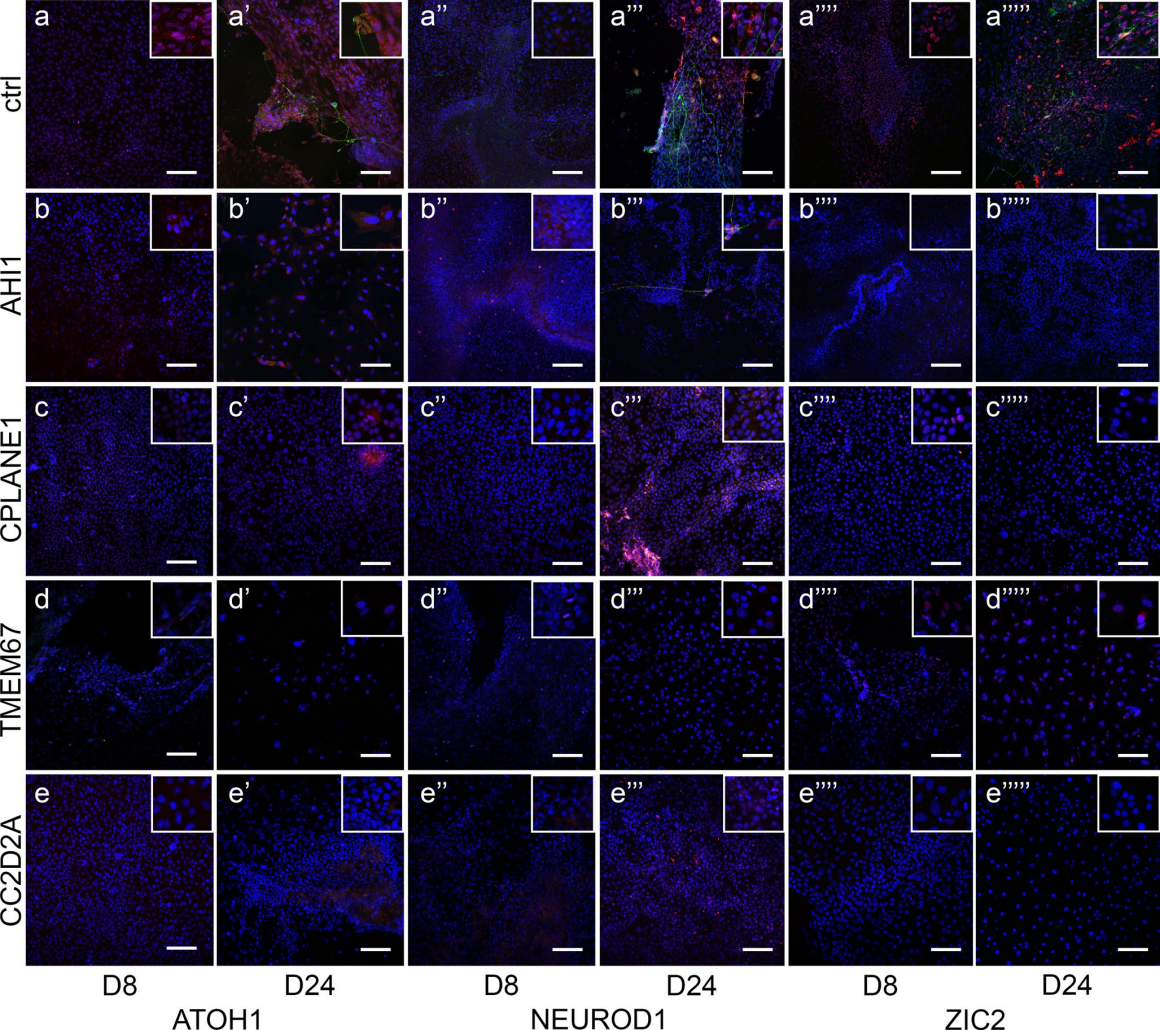
Differentiation of iPSCs towards mid-hindbrain and cerebellar lineages. Immunofluorescence co-staining for β3-TUBULIN (green) with the differentiation markers (red) ATOH1 (**a**-**e**, **a**’-**e**’), NEUROD1 (**a**’’–**e**’’, **a**’’’–**e**’’’) and ZIC2 (**a**’’’’–**e**’’’’, **a**’’’’’–**e**’’’’’) performed in CT-iPSCs (upper panel) and JS-iPSC lines (lower panels) after 8 and 24 days of differentiation (additional time points shown in Online Resource Fig. 11). Nuclei were counterstained with Hoechst (blue). Scale bar = 100 µm

**Fig. 3:**
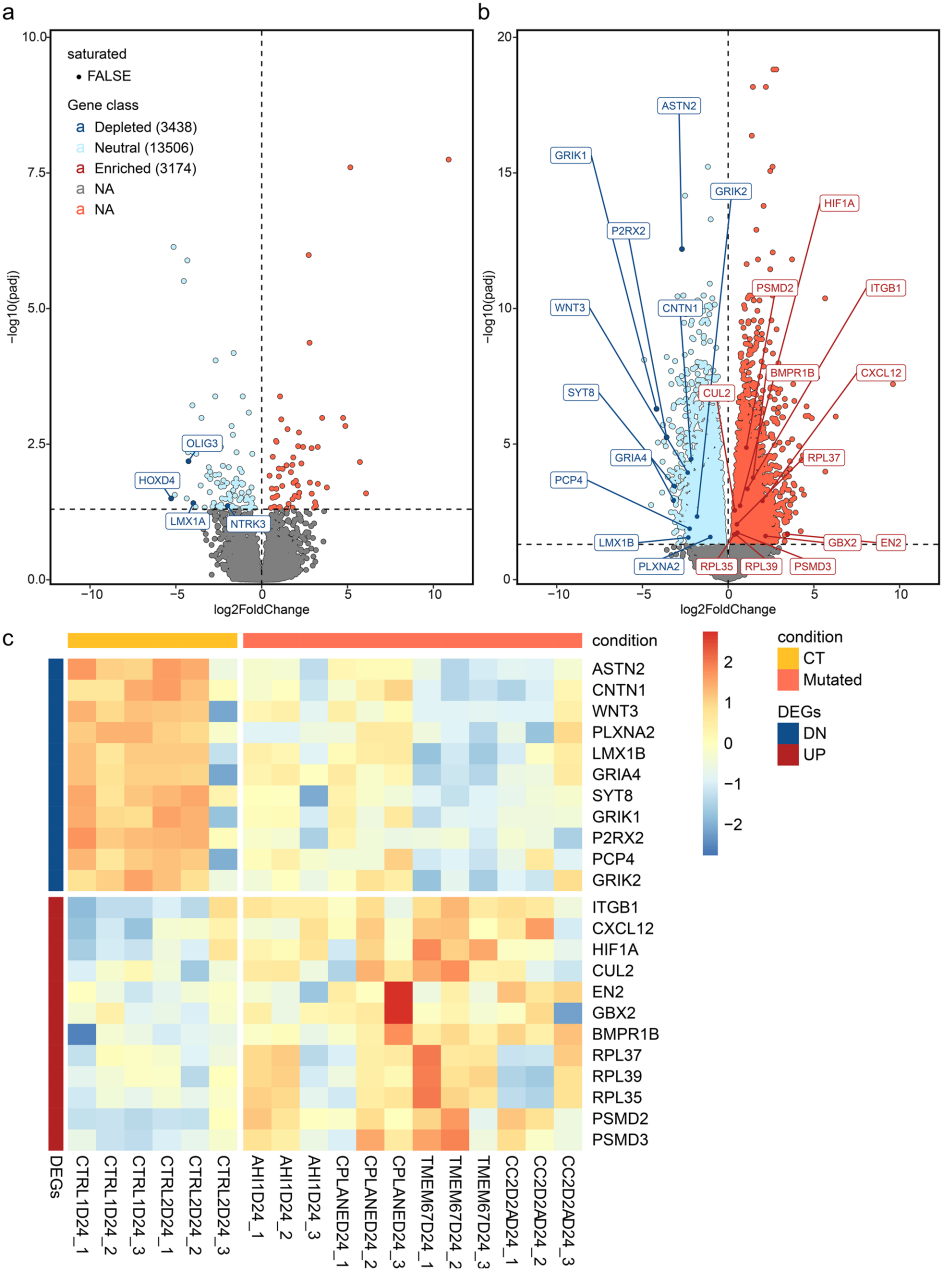
Transcriptomics analysis of differentially expressed genes in CT and JS iPSC lines. **a**, **b** Volcano Plots for T8 (**a**) and T24 (**b**) representing the logarithm of fold-change (in x) and the negative logarithm of the *p*-value (in y) of genes that are significantly regulated (color) determined by predefined thresholds for both fold-change and *p*-value. **c** Rpkm expression heatmap (in *z*-score) of selected genes

**Fig. 4:**
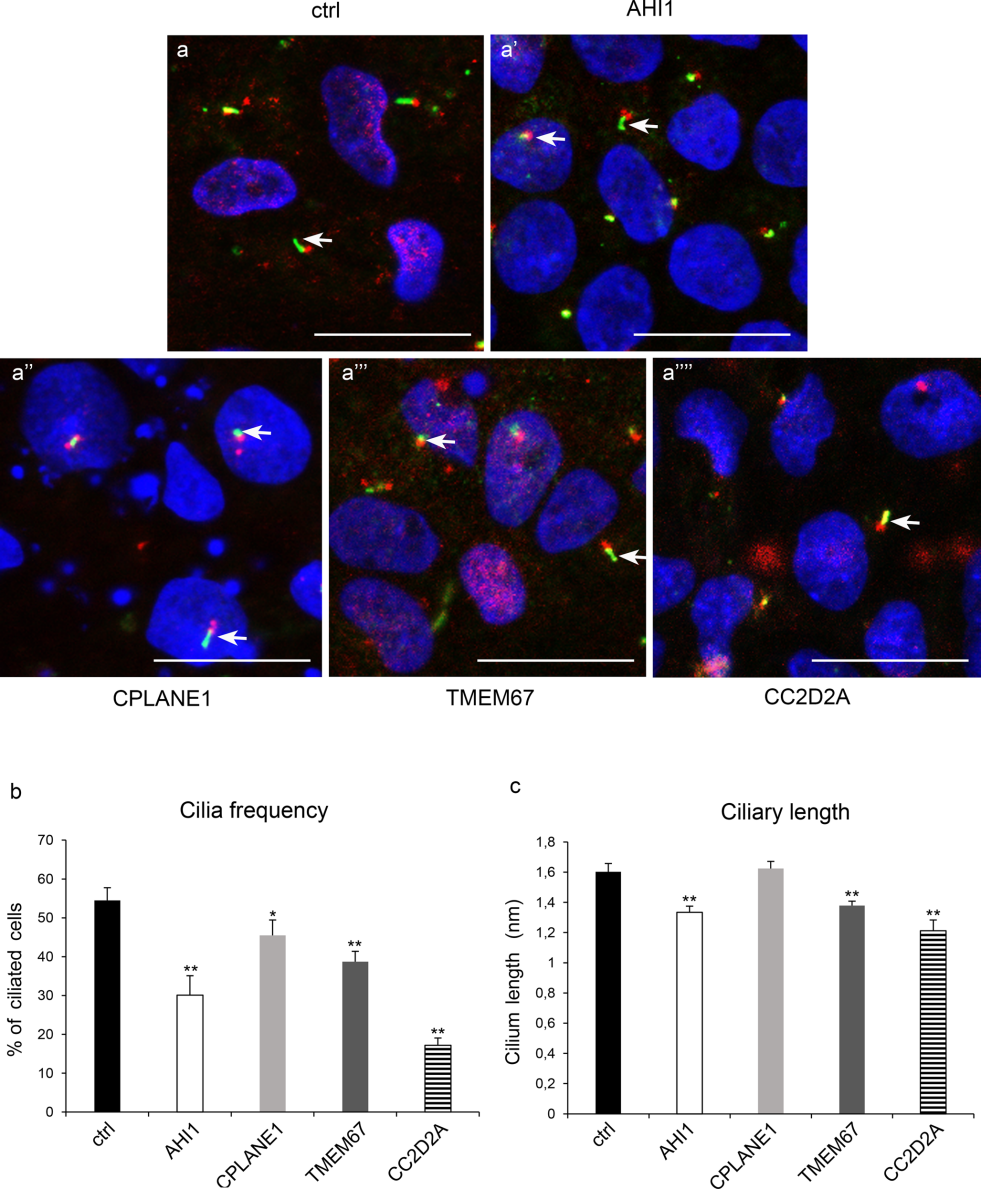
Analysis of primary cilia in control and JS-derived iPSCs at day 8 of differentiation. (**a**–**a**’’’’) Representative immunofluorescence images showing cilia labelled with anti-AcTUBULIN (green) and anti-PERICENTRIN (red) with Hoechst nuclear counterstain (blue) in each line. Scale bar: 20 µm. Lower magnification views shown in Online Resource Fig. 12. (**b**) Quantification of the mean proportion of ciliated cells in control and JS-iPSCs. (**c**) Analysis of ciliary length in control and JS-iPSCs. Results are presented as mean ± standard error of three independent experiments (*n* = 10 per sample). *p < 0.05; **p < 0.005

Dermal fibroblasts obtained from 2 healthy donors (CT-iPSCs, both young adult males) and 4 JS patients (JS-iPSCs, two males and two females, age range 10–27 years) carrying mutations in *AHI1*, *CPLANE1*, *TMEM67*, and *CC2D2A* (Table 1) were isolated and reprogrammed into iPSCs using the CytoTune™-iPS 2.0 Sendai Reprogramming Kit (Thermo Fisher Scientific). iPSC lines used for this study were produced by pooling of cells after reprogramming and characterized to confirm the expression of stemness markers and normal karyotype (Online Resources Figs. 1–8) as described in Online Resources. For all experiments, iPSCs were used at passages from 12 to 17.

**Table 1:** Biallelic variants carried by each JS-iPSC line

Gene	OMIM	Variant 1	Variant 2
*AHI1*	*608894	c.1123A > C; p.D666W	c.1997A > T; p.T375P
*CPLANE1*	*614571	c.3868 T > C; p.S1290P	c.C7477T; p.R2493X
*TMEM67*	*609884	c.755 T > C; p.M252T	c.1769 T > C; p.F590S
*CC2D2A*	*612013	c.3856 T > C; p.C1286R	c.3856 T > C; p.C1286R

### Differentiation of iPSCs into cerebellar granule cells

Differentiation of iPSCs towards mid-hindbrain precursors and GCs was performed using a previously published protocol (Erceg et al. 2012; Compagnucci et al. 2016) further optimized in the lab. Briefly, proliferating iPSCs were cultured as a monolayer on matrigel-coated dishes in StemFlex medium (Thermo Fisher Scientific RRID:SCR_018018) with Penicillin/Streptomycin until they reached 70% confluence. iPSCs were then switched to a differentiating medium containing specific cocktails of cytokines (BMPs, FGF family members, Shh and Wnt family members) added at specific time points to induce first mid-hindbrain precursors and later cerebellar granule cells after 31 days of culture (Online Resource Fig. 9).

### Immunofluorescence

iPSCs treated on coverslips were fixed in paraformaldehyde for 10 min, then permeabilized in PBS with 0.2% Triton X for 5 min, rinsed and blocked in PBS with 10% BSA prior to overnight incubation with the following primary antibodies: anti-ATOH1 (Abcam ab 85,513 RRID:AB_1924798), anti-NEUROD1 (Abnova PAB3857 RRID:AB_1575596), anti-ZIC2 (Millipore AB15392 RRID:AB_1977437), anti-β3-TUBULIN (Merck MAB1637 RRID:AB_2210524), followed by 1 h incubation with anti-rabbit Alexa fluor 555 and anti-mouse Alexa fluor 488 (1:5000) (Thermo Fisher Scientific). Nuclei were stained with Hoechst 33258 (Thermo Fisher Scientific). Images were analyzed with a confocal microscope (Zeiss). Ciliary count and length measurements were performed in iPSCs at the first differentiation time-point (D8). Cells at day 8 of differentiation were fixed in paraformaldehyde for 10 min then permeabilized in PBS with 0.2% Triton X for 5 min, rinsed and blocked in PBS with 10% BSA prior to overnight incubation with anti AcTUBULIN (Thermo Fisher Scientific cat 32-2700 RRID:AB_2533073) and anti-PERICENTRIN (Sigma HPA016820 RRID:AB_1855079), followed by 1 h incubation with rabbit Alexa fluor 555 and Anti mouse Alexa fluor 488 (1:5000). These experiments were performed in triplicate for each iPSC line, and cilia were measured and counted from 10 randomly selected fields on a microscope slide, counting a minimum of 100 cells per sample (n = 3 independent experiments) to calculate the percentage of ciliated cells (as the number of ciliated cells divided by the total number of cells). For ciliary length, the length from base to tip was analyzed with ImageJ (RRID:SCR_003070). The values shown for CT-iPSCs are the mean of the values for the two iPSC lines.

### qRT-PCR

Total RNA was extracted using the High Pure RNA Isolation Kit (Roche RRID:SCR_004098) and then reversely transcribed with SuperScript II Reverse Transcriptase (Thermo Fisher Scientific) according to the manufacturer’s instructions. The resulting cDNAs for 3 biological replicates (with 2 technical replicates each) were analyzed by real-time PCR using SYBR green on the Light Cycler 480 (Roche RRID:SCR_020502) using primers listed in Online Resource Table 2–4, with β*-ACTIN* used as internal control. The relative expression was calculated using the 2^ΔΔ^CT method. Relative fold change in marker gene expression was normalized to D0 for each line.

### Statistical analysis

Descriptive statistics for the proportion of cells able to make primary cilia ciliary counts and cilium length are calculated as mean and standard deviation while qRT-PCR data are calculated as median and interquartile range (IQR), given the non-normal distribution of most variables according to the Shapiro-Wilk test. Instead, for categorical variables, absolute and relative frequencies were calculated. To compare impaired ciliation in controls and JS-derived iPSCs, two-tailed *t*-tests were performed. To assess the differences between qRT-PCR groups, Kruskal-Wallis tests were performed and post-hoc comparisons of each group vs the control group were conducted using the non-parametric Wilcoxon-Mann-Whitney test. A *p*-value of < 0.05 was considered statistically significant. Bonferroni multiple testing correction was not applied to the post-hoc *p*-values due to the small sample size and the explorative nature of the analyses.

### Bulk transcriptomics

Total RNA was extracted from each cell line at day 8 and day 24 as described above. RNA quality and integrity, checked by RNA Integrity Number (RIN), were evaluated on an Agilent 2100 Bioanalyzer (Agilent Technologies, Santa Clara, CA, USA). Total RNA was sequenced with the Illumina NovaSeq 6000 technology in service at CeGaT GmbH (Tübingen, Germany) to obtain paired-end reads of 100 bp. At least 3 independent biological replicates were used for each line. Reads were trimmed with Trimmomatic (v0.39) (Bolger et al. 2014) to remove adapters and exclude low-quality reads from the analysis and aligned to the human reference genome using STAR aligner (v2.5.3a) (Dobin et al. 2013). FeatureCounts (v1.6.4) (Liao et al. 2014) was employed to calculate the reads mapping to exons based on the GENCODE human basic gene annotation. The resulting counts matrix represented transcript quantification summarized at the gene level, with the exclusion of reads mapping to multiple genomic locations. The counts matrix underwent a filtering process, retaining genes that exhibited a minimum of 1 CPM (counts per million) in at least 9 samples. Differential gene expression analysis was performed with DESeq2 (Love et al. 2014) bioconductor library (v1.30.1). The rpkm values were computed with edgeR (v3.40.2) bioconductor library (Robinson et al. 2010). Genes with statistically significant expression changes were identified using the following criteria: padj < 0.03, log2 fold change > 1, and log2 fold change <  − 0.3. Heatmaps were made with ComplexHeatmap R package (v2.11.1) (Gu et al. 2016). Visualization of differentially expressed genes was achieved by creating a Volcano Plot using the ggplot2 package in R (v3.4.4). Transcriptomics data files are available on GEO (accession number: GSE254556) and analysis scripts are available on Zenodo (10.5281/zenodo.10355929).

## Results

### iPSCs can be differentiated towards mid-hindbrain precursors and cerebellar neurons

CT-iPSCs (Ali et al. 2021) were first used to optimize the differentiation protocol to produce mid-hindbrain precursors and then cerebellar granule cells (CGCs), as previously reported (Erceg et al. 2012; Compagnucci et al. 2016). Differentiation was assessed every 7 days from day 0 (proliferating cells) to day 31 through qRT-PCR analysis of the differentiation markers *ATOH1*, *NEUROD1*, and *ZIC2* (Fig. 1a–c). As expected, both CT-iPSC lines expressed these markers according to the sequential differentiation steps induced by the protocol (Online Resource Fig. 9). The expression of the early neurogenic marker *ATOH1* was already evident at day 8, steadily increased till day 24, and remained high in both lines until the end of the protocol. The expression of *NEUROD1*, a transcription factor promoting cerebellar differentiation, was induced after 2 weeks and remained highly expressed until day 31. *ZIC2*, which is expressed in pluripotent cells and is maintained in granule neuron precursors (Luo et al. 2015), was detected throughout the protocol.

To further substantiate these observations, two additional neural genes important for the correct development and organization of mature cerebellum, *PAX6*, and *LMX1B* were monitored (Fig. 1d, e). In line with previous observations (Luo et al. 2015), CT-iPSCs showed a progressive increase of PAX6 levels at early time points, followed by a decrease upon lineage maturation. *LMX1B*, a key gene for midbrain and cerebellum development, was detected by D8 in CT-iPSCs with a strong increase after D24.

### Cerebellar differentiation is impaired in iPSCs derived from JS patients

To evaluate how disruption of distinct JS-associated genes may impact on neuronal differentiation, iPSCs were generated from primary fibroblasts of four JS patients carrying pathogenic variants in either *AHI1*, *CPLANE1*, *TMEM67*, or *CC2D2A* (Table 1).

These iPSC lines (JS-iPSCs) were validated for pluripotency marker expression and karyotype integrity according to standard procedures (Ali et al. 2021). JS-iPSCs were treated according to the differentiation protocol validated in CT-iPSCs, and the expression of differentiation markers was analyzed by qRT-PCR over the 31-day time-course. D31 analysis could only be performed on *CPLANE1* JS-iPSCs due to the consistent cell loss experienced by the other JS-iPSCs lines over the last week of differentiation (after D24).

All JS-iPSC lines showed altered expression profiles for the selected differentiation markers compared to control cells (Fig. 1). Expression levels of *ATOH1* transcript were consistently lower in all JS lines compared to CT-iPSCs at D8 and D16, and at D24 for *CPLANE1*, *AHI1*, and *TMEM67* lines. Similarly, *NEUROD1* transcript levels were lower in patient lines particularly at D8 and D16, but significatively so at D24 for the *AHI1* line. *ZIC2* expression was not detected in *AHI1* and *CC2D2A* JS-iPSCs, whereas it was present but lower than controls in *CPLANE1* and *TMEM67*-mutated lines, especially at D24. Moreover, *LMX1B* mRNA levels were significantly lower in the *TMEM67* line compared to controls at D16 and D24, while *PAX6* expression increased in JS-iPSCs lines along the differentiation protocol, although with some variability across different mutant lines (Online Resource Table 1). Principal component analysis of D14 expression levels confirmed the clear difference in marker expression profile between differentiated controls and clustered mutant lines (Online Resource Fig. 10).

To confirm the altered mRNA expression profile seen in JS-iPSCs, immunofluorescence analysis was performed to investigate the expression of ATOH1*,* NEUROD1, and ZIC2 proteins in JS patient cells and in healthy controls over the differentiation treatment. In keeping with the qRT-PCR results, ATOH1, NEUROD1, and ZIC2 proteins were either reduced compared to controls (for *AHI1* and *CPLANE1* lines), or undetectable (for *TMEM67* and *CC2D2A* lines) (Fig. 2 lower panels and Online Resource Fig. 11). Immunostaining for β3-TUBULIN, a microtubule protein of the tubulin family marking neurons, showed a well-defined network of positive cells in CT-iPSCs, particularly at D31 of differentiation (Online Resource Fig. 11). In contrast, JS-iPSCs showed rare branching of β3-TUBULIN-positive neurons (Fig. 2 arrows and Online Resource Fig. 11).

In light of these observations, a differential gene expression analysis was performed on total RNA extracted from D8 and D24 for each differentiated iPSC line to survey a larger number of marker genes. Differentially expressed genes (DEGs) between CT and JS lines were analyzed at both timepoints (Fig. 3). Results show differences in the expression already at D8, with genes relating to the central nervous system development and in particular cerebellum markers (*LMX1A*, *OLIG3*) (Consalez et al. 2021; Lowenstein ED et al. 2021) being expressed only in controls cells. At D24, differences in gene expression became more evident, underlining for CT samples the expression of genes specific to the terminal phases of cerebellar differentiation (*ASTN2*, *CNTN1*, *WNT3*, *PLXNA2*, *LMX1B*) (Consalez et al. 2021) and the expression of genes encoding proteins of neuronal functionality (*GRIA4*, *SYT8*, *GRIK1*, *P2RX2*, *PCP4*, *GRIK2*) (Hansen et al. 2021; Rubio et al. 2001; Harashima et al. 2011). In contrast, the expression of these genes was significantly lower in JS lines, which showed early differentiation markers still predominantly expressed (*ITGB1*, *CXCL12*, *HIF1A*, *EN2*, *GBX2*, *BMPR1B*, *CUL2*, *RPL37*, *RPL39*, *RPL35*, *PSMD2*, *PSMD3*) (Consalez et al. 2021; Gilthorpe et al. 2002). Taken together, these data support the previous observation that JS-iPSC lines show an impaired progression along the differentiation time course and a reduced ability to reach the maturation state seen in controls.

### iPSCs derived from JS patients show impaired ciliation and reduced length of primary cilia

The proportion of cells able to make primary cilia and the relative ciliary length was compared in control and JS-derived iPSCs at day 8 of differentiation by immunofluorescence staining using acetylated TUBULIN (AcTUBULIN) and PERICENTRIN as markers of the ciliary axoneme and basal body, respectively (Fig. 4a–a’’’’ and Online Resource Fig. 12). The percentage of ciliated cells in all JS-derived iPSCs was significantly lower compared to healthy controls, although to various degrees (control 54.5 ± 3.3%; AHI1 20.1 ± 5% (*p* < 0.005); CPLANE1 45.5 ± 3.9% (*p* < 0.05); TMEM67 38.7 ± 2.7% (*p* < 0.005); CC2D2A 17.1 ± 1.9% (*p* < 0.005)) (Fig. 4b). Three JS lines (AHI1, TMEM67, CC2D2A) also showed a significant reduction of ciliary length, while no significant change in length was observed in CPLANE1 mutant cells compared to controls (control 1.6 ± 0.05 µm; AHI1 1.3 ± 0.04 µm; CPLANE1 1.6 ± 0.04 µm; TMEM67 1.4 ± 0.03 µm; CC2D2A 1.2 ± 0.07 µm, (*p* < 0.005 for all comparisons) (Fig. 4c).

## Discussion

Joubert syndrome (JS) is characterized by a unique cerebellar and brainstem malformation, the MTS, which is linked to a multiplicity of genetic defects. No therapies or modifying treatments are currently available for this disorder (Rosati et al. 2018). All the > 40 JS-linked genes encode proteins of the primary cilium, a subcellular organelle widely present in embryonic and adult cells. To date, however, it remains unclear how disruption of distinct proteins with diverse roles in the structure or functioning of the cilium may eventually result in such a specific and peculiar defect as the MTS. In this light, iPSCs represent a simple and efficient model to study abnormal neuronal development in ciliopathies such as JS. To date, a few studies have reported the generation of iPSCs carrying mutations in JS-related genes, including *AHI1* (Rosati et al. 2018; Altieri et al. 2019; Serpieri et al. 2023), *CPLANE1* (Ali et al. 2020), *CC2D2A* (Ali et al. 2021), *CEP290* (Shimada et al. 2017; May-Simera et al. 2018), *RPGRIP1L* (Wang et al. 2019), and *TMEM67* (Barabino et al. 2020); however, these studies did not systematically analyze differentiation markers or compare ciliary phenotypes. The present study employed iPSCs derived from patients carrying pathogenic variants in four of the five common genes causative of JS, which were differentiated towards mid-hindbrain precursors and cerebellar neurons in order to address two main research questions: (1) do JS-derived iPSCs show aberrant differentiation along the cerebellar lineage? (2) Are there any patent differences among cells mutated in distinct genes?

Interestingly, the four iPSC lines revealed shared ciliary and differentiation alterations in vitro. While differentiated CT-iPSCs correctly expressed markers for mid-hindbrain and cerebellar granule cell precursors along the in vitro time-course, JS mutant cells showed a marked alteration in their differentiation capacity. A progressive cell loss was observed in three of the JS-iPSC lines (*AHI1*, *TMEM67*, *CC2D2A*) during the last phase of differentiation treatment (post D24), and all JS-iPSC lines showed a reduced formation of β3-TUBULIN-positive neurons, suggesting a lower differentiation capacity. This in vitro response was reminiscent of observations made for iPSC lines from other ciliopathies (such as Meckel syndrome and Bardet-Biedl syndrome), reporting increased cell loss in culture and differentiation delays (Barabino et al. 2020). The present observation warrants further studies to assess possible defects linked to apoptosis and autophagy pathways, two key mechanisms already linked to ciliogenesis (Attanasio et al. 2007; Tang et al. 2013; Morleo and Franco 2019) and neural differentiation (Mazarakis et al. 1997; Rosiak et al. 2016) and therefore potentially affected in JS-derived cultures. The mRNA expression levels for two genes finely regulated during neural development, *LMX1B*, and *PAX6*, were severely misregulated in JS cells. *LMX1B* is a master gene for the development and maintenance of the isthmic organizer (Mishima et al. 2009; Srivastava et al. 2017), which is thought to secrete planar signals regulating the development of the mid/hindbrain (Shimada et al. 2017). *LMX1B* expression in all JS-derived cultures was observed to be lower than controls, suggesting a failure of the mutant lines to adequately activate a midbrain/cerebellar differentiation program. In contrast, transcriptional levels of the neuroprogenitor marker *PAX6* in JS-iPSCs failed to show the typical differentiation-related reduction seen in CT-iPSCs (Erceg et al. 2010; Ha et al. 2012). The JS-iPSC lines analyzed here also demonstrated an altered expression of the mid-hindbrain markers *ATOH1* and *NEUROD1*, which are both required for the formation of the granule cell population. *ATOH1* regulates early neuronal differentiation leading to the formation of CGC precursors, and its deletion results in the elimination of the granule cell population (Brown and Witman 2014; Lange et al. 2021). *NEUROD1* acts subsequently to promote the differentiation of CGC precursors towards the mature cerebellar granule cell lineage (Pan et al. 2009); it is also involved in the maintenance of this differentiated population, as evidenced by the increased cell death detected in *NeuroD1* knockout models (Warburton-Pitt et al. 2012). In light of this, it is tempting to speculate that the observed reduction of *NEUROD1* expression may somehow relate to the massive cell loss experienced by three mutant cell lines. Finally, JS-iPSCs showed reduced *ZIC2* expression during the later phase of the differentiation protocol, in particular by D24, as also observed at the protein level. *ZIC2* is reportedly expressed in pluripotent cells (Brown and Brown 2009), while in differentiated lineages it is a marker of granule cell precursors, acting together with *ZIC1* to control cerebellar development (Schouteden et al. 2015; Bashford and Subramanian 2019). In support of these observations, the transcriptomics analysis underlined the reduced mature marker expression in JS lines. Compared to controls, JS lines showed a profile more typical of early phases of differentiation. Taken together, this altered expression profile in JS-iPSC cultures appears consistent with an inadequate activation of genes controlling neuronal/cerebellar differentiation, a situation not dissimilar to that seen in iPSCs from patients with Bardet-Biedl syndrome mutated in *BBS10*, which responded to neural crest differentiation with a lower neural crest genes expression and incomplete differentiation (Barrell et al. 2019). Of note, alterations detected at the mRNA level for neuronal markers were largely echoed by immunostaining observations; it will be useful to further confirm these quantitatively, for instance by performing a Western blot analysis, and extend the analysis to glial markers to assess whether additional lineages may contribute to the phenotype. Furthermore, experiments on larger samples of iPSCs from JS patients will address whether distinct JS mutations in the same gene will result in a similar differentiation impairment.

iPSCs possess a primary cilium which becomes more evident in the course of neuronal differentiation (Rosenbaum and Witman 2002; Wood et al. 2013) and are thus a fitting model to study cellular defects in ciliopathies caused by different genetic mutations. Among the four JS patient lines generated here, a significant reduction in the proportion of mutant iPSCs able to form the primary cilium was consistently observed compared to control cells. Ciliary measurements further indicated a reduced cilium length that was significant in three out of the four JS-iPSC lines (*AHI1*, *TMEM67*, *CC2D2A*), while results for the *CPLANE1* line were similar to those of control cells. These results align with previous reports on fibroblasts from patients mutated in either *CSPP1*, *ARL13B*, and *CEP41*, describing cellular defects in cilium morphology, number or defects in the ciliary axoneme (Tuz et al. 2014; Ramsbottom et al. 2018). Barabino et al. also reported abnormal ciliary phenotype in a *TMEM67*-mutant iPSC line, although in this case more numerous, but shorter and thinner cilia were observed, suggesting that different mutations in the same JS gene can lead to different outcomes. Moreover, iPSCs from *RPGRIP1L*-mutated JS patients showed a reduction in the number of ciliated neurons formed upon differentiation (Altieri et al. 2019), while JS iPSCs mutated in *CEP290*, when differentiated towards retinal epithelium lineages, showed smaller cilia associated with incomplete lineage maturation (Rosati et al. 2018). Further in vitro observations made in other ciliopathy models, such as another iPSC line from a *CEP290*-mutated patient with Leber’s congenital amaurosis and differentiated towards retinal pigment epithelium, also showed alterations in primary cilium formation and incidence (Parfitt et al. 2016). Of note, in our experimental setting, the *CPLANE1* line was the only line to survive over the differentiation time-course and showed a normal ciliary length with less evident differences from controls regarding the proportion of ciliated cells. This observation contrasts with previous observations made on *CPLANE1* mutant fibroblasts, which showed fewer and shorter primary cilia and diminished response to a SHH agonist compared to controls (Asadollahi et al. 2018). This would call for further analysis in a larger number of *CPLANE1* iPSC lines carrying distinct mutations, as we cannot exclude an hypomorphic effect of the *CPLANE1* missense variant p. S1290P, potentially resulting in a less detrimental effect on the protein function, as already reported for other variants in distinct JS-related genes (Leitch et al. 2008). Additional investigations including a longitudinal cilium analysis over the cerebellar differentiation time-course, a spatial characterization of the ciliary position for the protein products of the 4 mutated genes (*AHI1*, *CPLANE1*, *TMEM67*, and *CC2D2A*), and a precise analysis of how different JS mutations impact on the functioning of the corresponding protein at the ciliary level will help to further understand the pathogenic mechanisms underlying the neurodevelopmental defects observed in patients’ cell lines. More generally, since some mutations in the JS-associated genes analyzed here have been associated with other ciliopathy phenotypes such as nephronophthisis and Meckel syndrome (Van De Weghe et al. 2022), it will be interesting to compare iPSCs lines from patients with different ciliopathy phenotypes harbouring mutations within the same gene to explore how these result in defects affecting different organs.

### Supplementary Information

Below is the link to the electronic supplementary material.

Supplementary file1 (DOCX 12807 KB)
