# Supplementary material for: Joubert syndrome-derived induced pluripotent stem cells show altered neuronal differentiation in vitro
Source: Cell Tissue Res. 2024 Mar 19;396(2):255–67. doi: 10.1007/s00441-024-03876-9 (PMC11055696; doi:10.1007/s00441-024-03876-9)
Supplement: Supplementary file 1 — Supplementary file1 (DOCX 12807 KB) [file 441_2024_3876_MOESM1_ESM.docx]

Supplementary Material

**Joubert Syndrome-derived induced pluripotent stem cells show altered neuronal differentiation *in vitro.***

Roberta De Mori ^1,†^, Silvia Tardivo ^2,†^, Lidia Pollara ^3^, Silvia Clara Giliani ^4^, Eltahir Ali ^4^, Lucio Giordano ^5^, Vincenzo Leuzzi ^6^, Rita Fischetto ^7^, Blanca Gener ^8^, Santo Diprima ^9^, Marco Morelli ^9^, Maria Cristina Monti ^10^, Virginie Sottile ^11,^* and Enza Maria Valente ^11,3,^*

^1^ Induced Pluripotent Stem Cells Unit, IRCCS Santa Lucia Foundation, Rome, Italy; r.demori@hsantalucia.it

^2^ Neurogenetics Lab, IRCCS Santa Lucia Foundation, Rome, Italy; silvia.tardivo@gmail.com

^3^ Neurogenetics Research Unit, IRCCS Mondino Foundation, Pavia, Italy; lidia.pollara01@universitadipavia.it; enzamaria.valente@unipv.it

^4^ Department of Molecular and Translational Medicine, University of Brescia, Brescia, Italy; silvia.giliani@unibs.it ; e.ali@unibs.it

^5^ Paediatric Neurology and Psychiatry Unit, Spedali Civili Children's Hospital, University of Brescia, Brescia, Italy; lucio.giordano@asst-spedalicivili.it

^6^ Unit of Child Neurology and Psychiatry, Department of Human Neuroscience, University of Rome La Sapienza, Rome, Italy; vincenzo.leuzzi@uniroma1.it

^7^ Clinical Genetics Unit, Department of Pediatric Medicine, Giovanni XXIII Children's Hospital, Bari, Italy; rfischetto@libero.it

^8^ Cruces University Hospital, Department of Genetics, BioBizkaia Health Research Institute, Cruces Plaza, 48903 Barakaldo, Bizkaia, Spain; blanca.generquerol@osakidetza.eus

^9^ IRCCS San Raffaele Hospital, Milan, Italy; Diprima.Santo@hsr.it; morelli.marco@hsr.it

^10^ Unit of Biostatistics and Clinical Epidemiology, Department of Public Health, Experimental and Forensic Medicine, University of Pavia, Pavia, Italy; cristina.monti@unipv.it

^11^ Department of Molecular Medicine, University of Pavia, Pavia, Italy; virginie.sottile@unipv.it


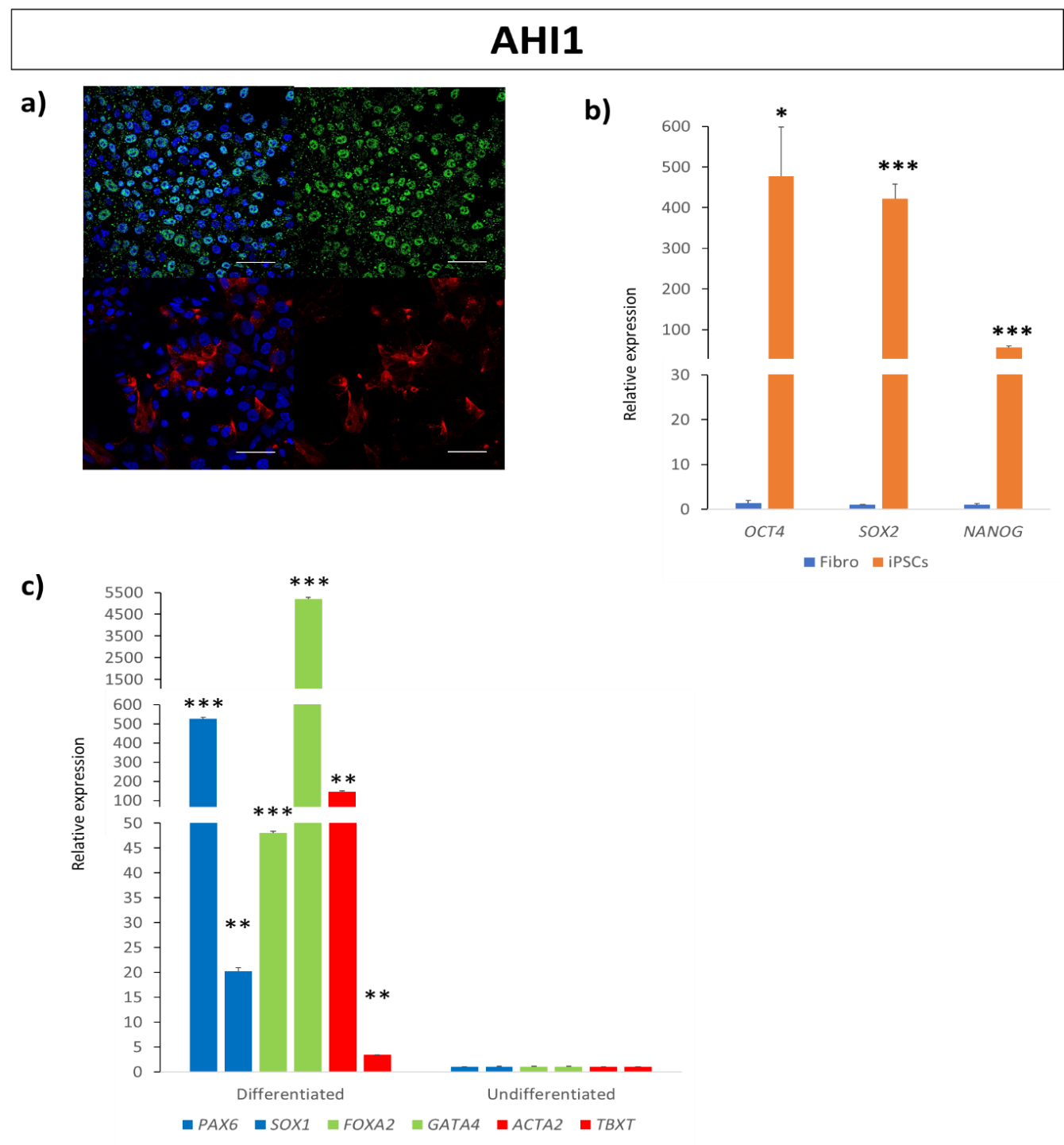


**Online Resource Fig.1.** Characterisation of the AHI1 iPSC line. **(a)** Immunofluorescence of stemness markers OCT4 (green), TRA-1-60 (red). Nuclei were counterstained with Dapi (blue). Scale bar = 50 mm. **(b)** qRT-PCR of stemness markers OCT4, SOX2 and NANOG. Data were normalized to ACTB and calculated in relation to parental fibroblasts. *P < 0.05; **P< 0.005; ***P< 0.0005; ****P< 0.00005. **(c)** qRT-PCR of germ layers markers: ectoderm (PAX6, SOX1), endoderm (FOXA2, GATA4) and mesoderm (ACTA2, TBXT). Data were normalized to ACTB. *P < 0.05; **P< 0.005; ***P< 0.0005; ****P< 0.00005.


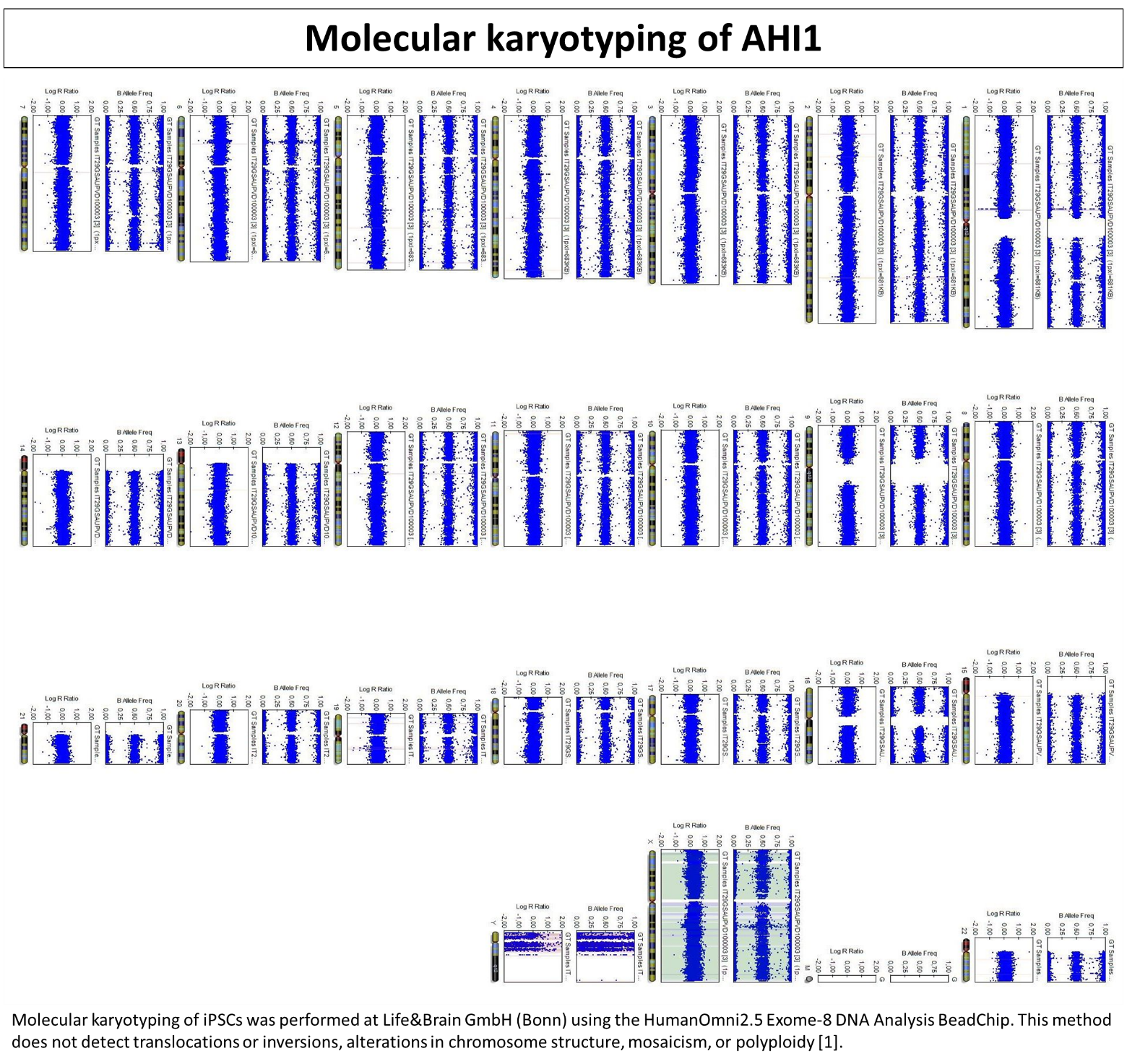


**Online Resource Fig.2.** Molecular karyotyping of AHI1 iPSC line, indicating the absence of pathogenic copy number variations.

..


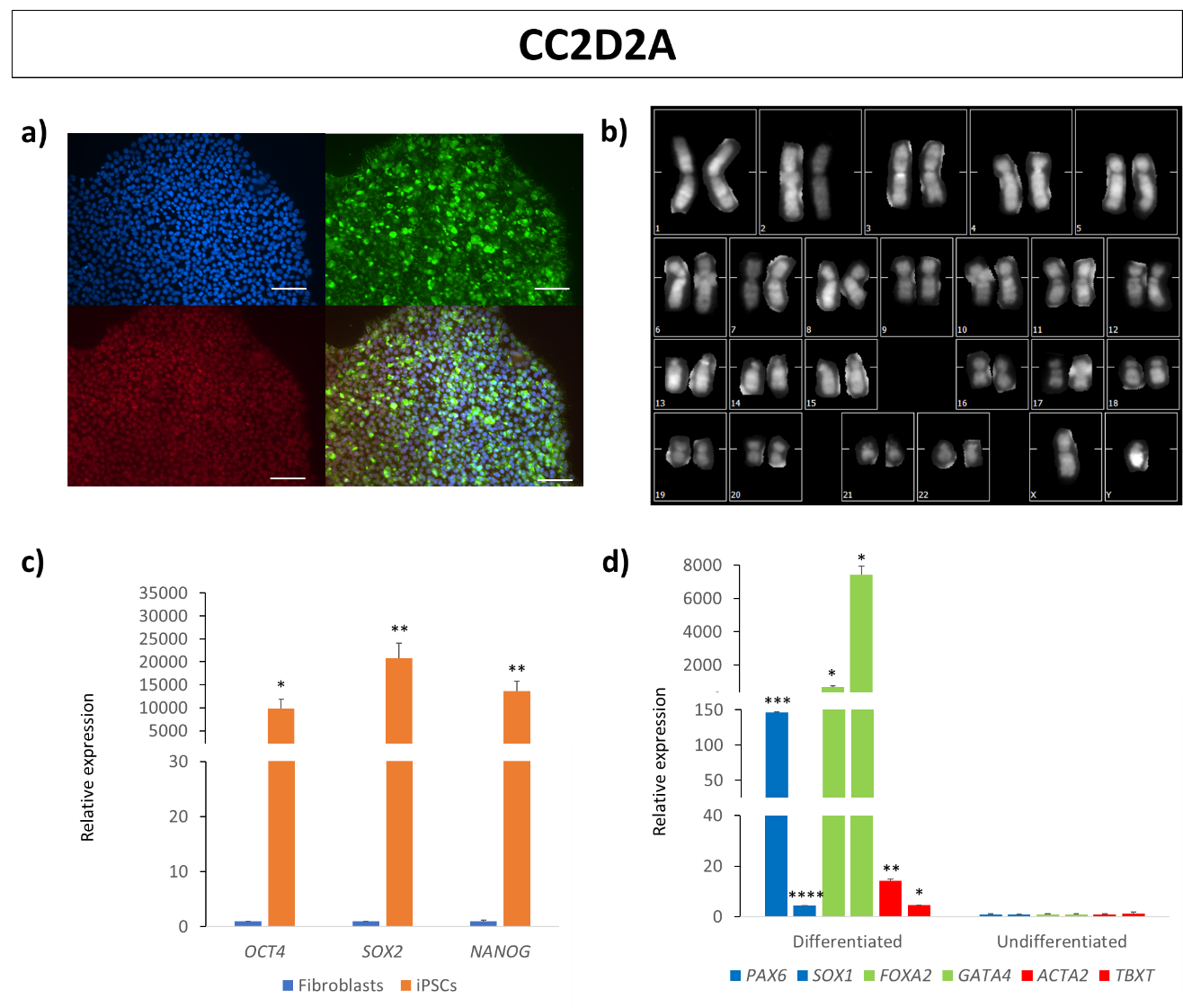


**Online Resource Fig.3.** Characterisation of the CC2D2A iPSC line. **(a)** Immunofluorescence of stemness markers OCT4 (red), TRA-1-60 (green). Nuclei are counterstained with Hoechst33342 (blue). Scale bar = 100 μm. **(b)** Karyotype, 46 XY. **(c)** qRT-PCR of stemness markers OCT4, SOX2 and NANOG. Data were normalized on ACTB and calculated in relation to parental fibroblasts. *P < 0.05; **P< 0.005; ***P< 0.0005; ****P< 0.00005. **(d)** qRT-PCR for germ layers markers: ectoderm (PAX6, SOX1), endoderm (FOXA2, GATA4) and mesoderm (ACTA2, TBXT). Data were normalized on ACTB. *P < 0.05; **P< 0.005; ***P< 0.0005; ****P< 0.00005.


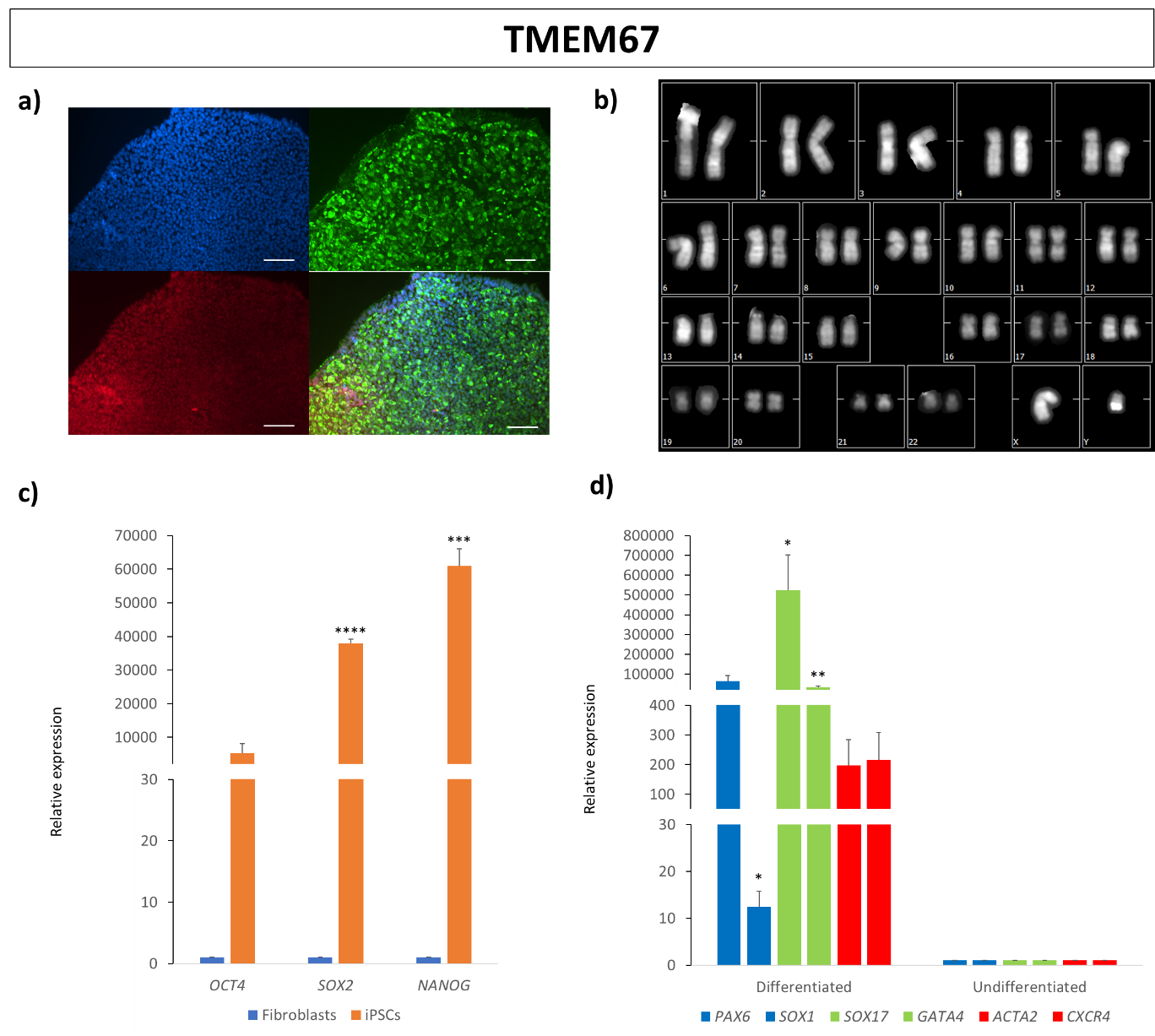


**Online Resource Fig.4.** Characterisation of the TMEM67 iPSC line. **(a)** Immunofluorescence of stemness markers OCT4 (red), TRA-1-60 (green). Nuclei are counterstained with Hoechst33342 (blue). Scale bar = 100 μm. **(b)** Karyotype, 46 XY. **(c)** qRT-PCR of stemness markers OCT4, SOX2 and NANOG. Data were normalized on ACTB and calculated in relation to parental fibroblasts. *P< 0.05; **P< 0.005; ***P< 0.0005; **** P< 0.00005. **(d)** qRT-PCR of germ layers markers: ectoderm (PAX6, SOX1), endoderm (SOX17, GATA4) and mesoderm (ACTA2, CXCR4). Data were normalized on ACTB. *P < 0.05; **P< 0.005; ***P< 0.0005; ****P< 0.00005.


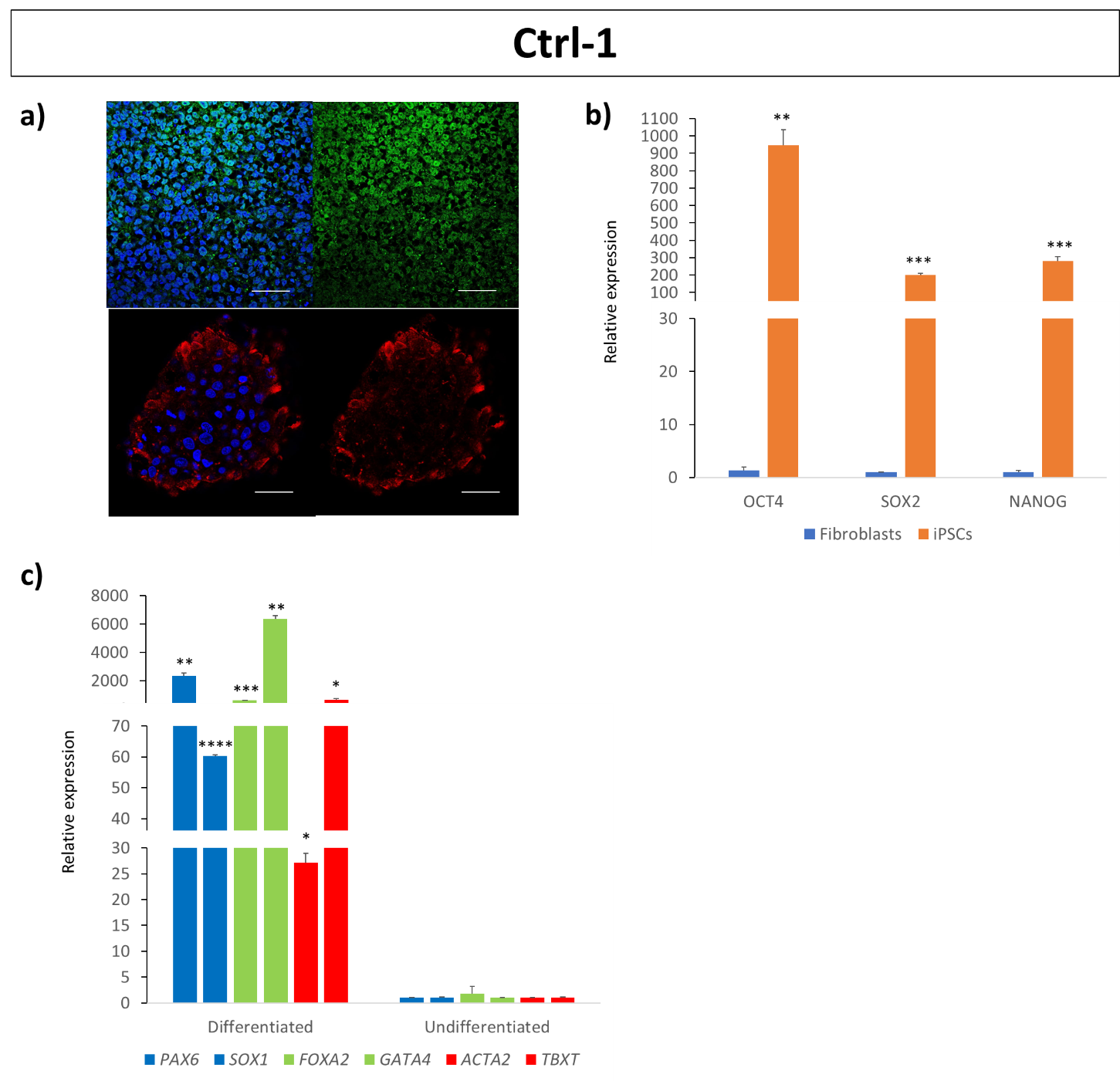


**Online Resource Fig.5.** Characterisation of the Ctrl-1 iPSC line. **(a)** Immunofluorescence of stemness markers OCT4 (green), TRA-1-60 (red). Nuclei are counterstained with DAPI (blue). Scale bar = 50 μm. **(b)** qRT-PCR of stemness markers OCT4, SOX2 and NANOG. Data were normalized on ACTB and calculated in relation to parental fibroblasts. *P < 0.05; **P< 0.005; ***P< 0.0005; **** P< 0.00005. **(c)** qRT-PCR of germ layers markers: ectoderm (PAX6, SOX1), endoderm (FOXA2, GATA4) and mesoderm (ACTA2, TBXT). Data were normalized on ACTB. *P< 0.05; **P< 0.005; ***P< 0.0005; ****P< 0.00005.


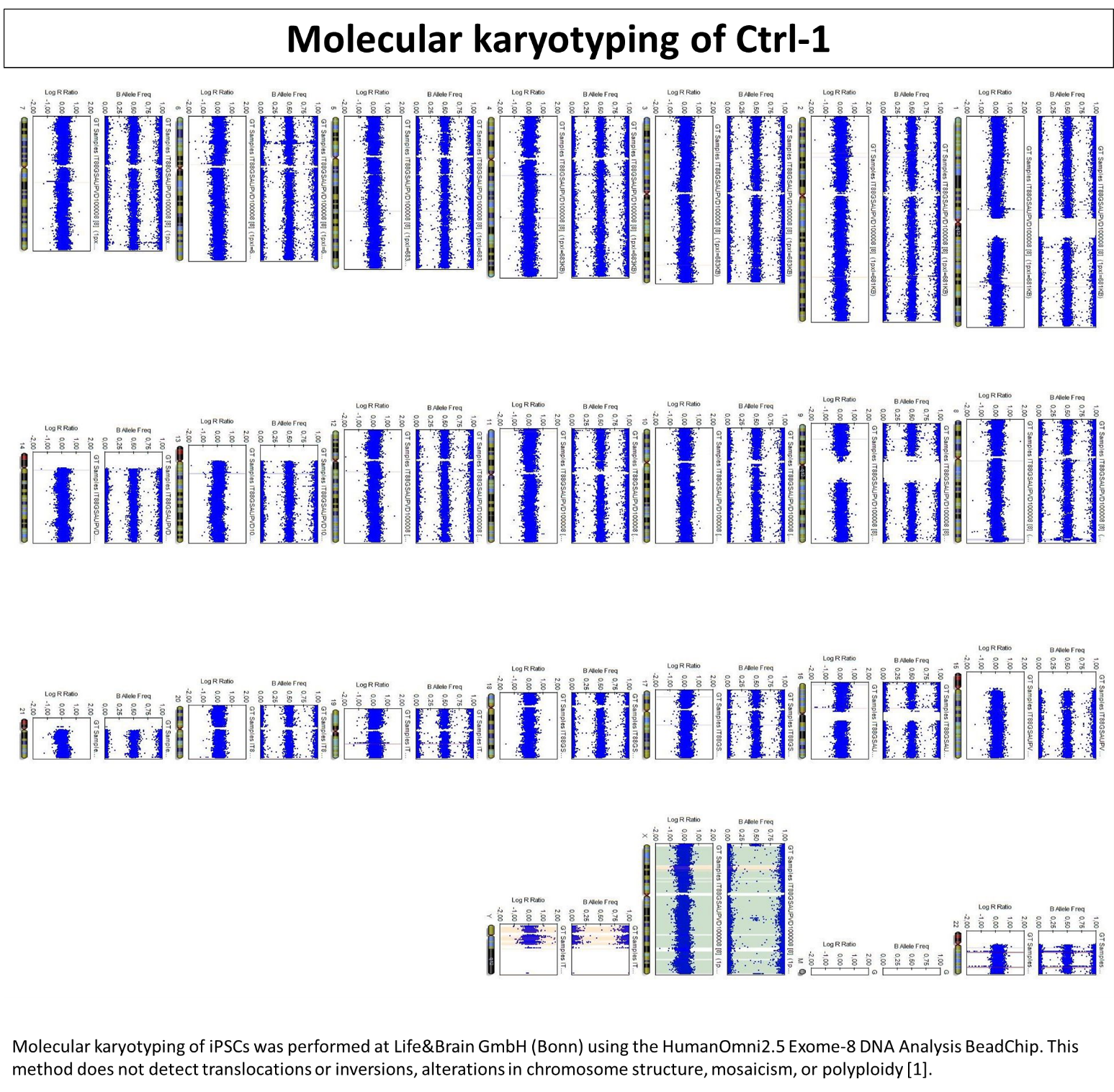


**Online Resource Fig.6.** Molecular karyotyping of Ctrl-1 iPSC line, indicating the absence of pathogenic copy number variations.


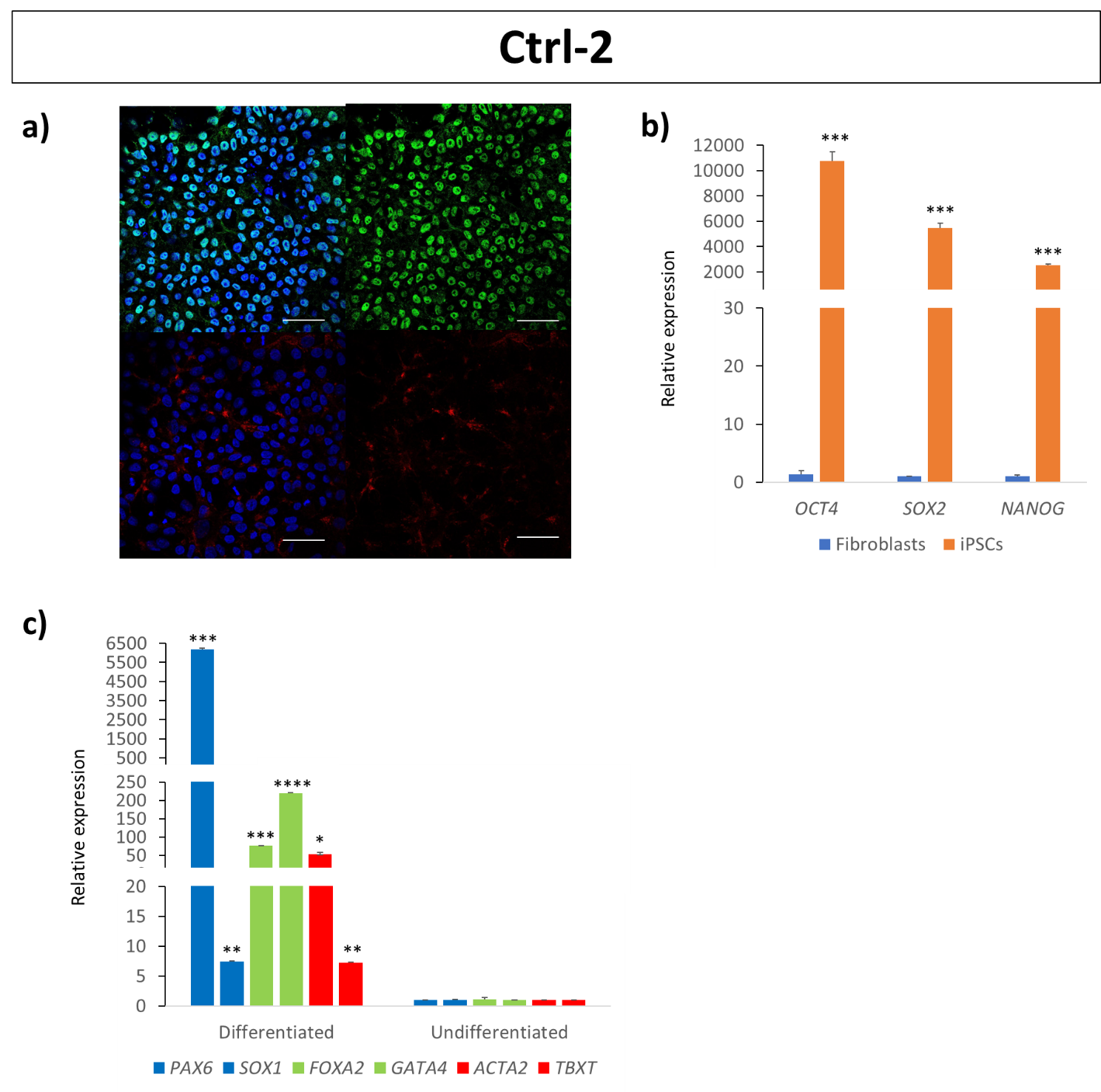


**Online Resource Fig.7.** Characterisation of the Ctrl-2 iPSC line. **(a)** Immunofluorescence of stemness markers OCT4 (green), TRA-1-60 (red). Nuclei are counterstained with DAPI (blue). Scale bar = 50 μm. **(b)** qRT-PCR of stemness markers OCT4, SOX2 and NANOG. Data were normalized on ACTB and calculated in relation to parental fibroblasts. *P< 0.05; **P< 0.005; ***P< 0.0005; ****P< 0.00005. **(c)** qRT-PCR of germ layers markers: ectoderm (PAX6, SOX1), endoderm (FOXA2, GATA4) and mesoderm (ACTA2, TBXT). Data were normalized on ACTB. *P < 0.05; **P< 0.005; ***P< 0.0005; ****P< 0.00005.


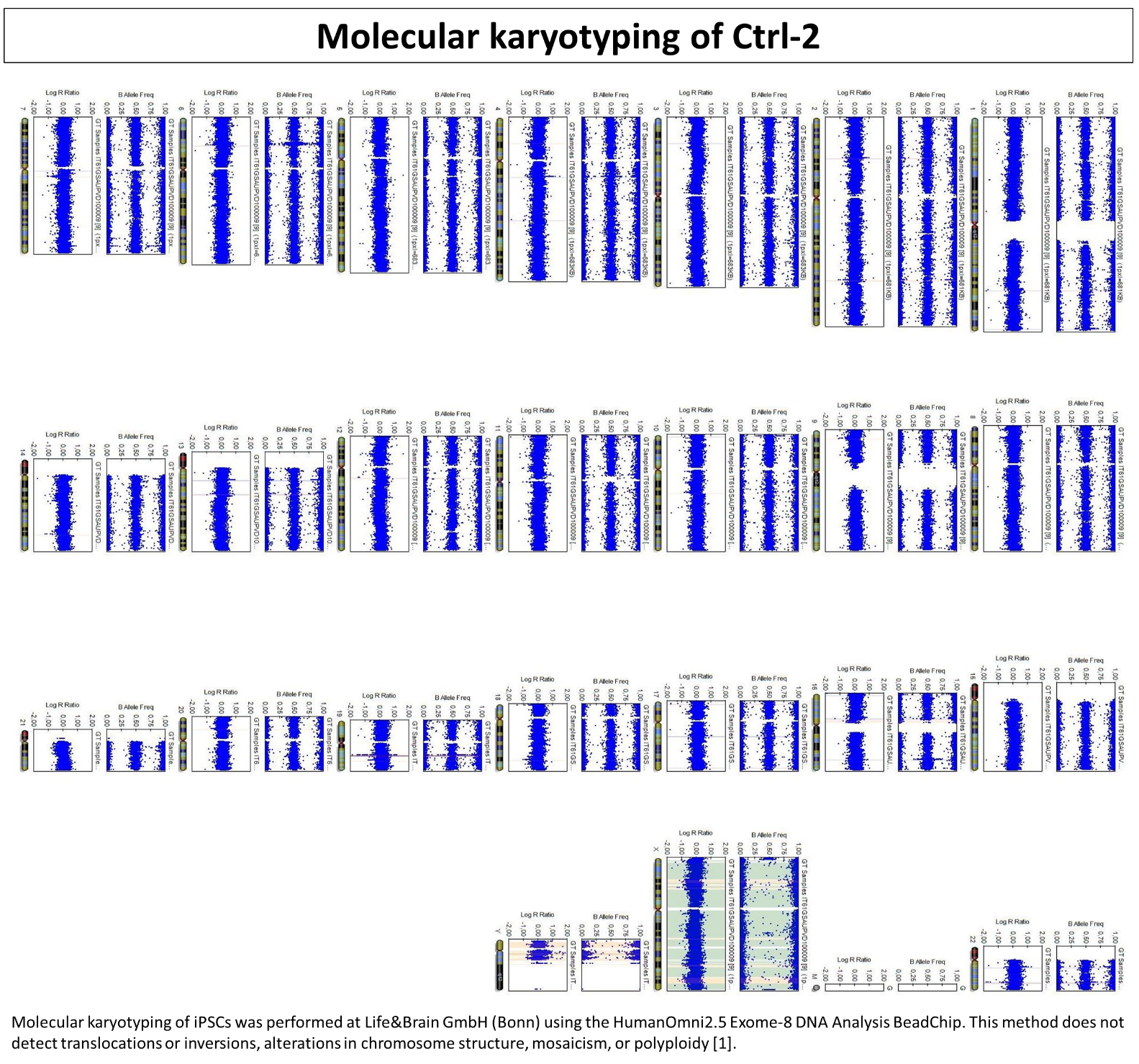


**Online Resource Fig.8.** Molecular karyotyping of Ctrl-2 iPSC line, indicating the absence of pathogenic copy number variations.


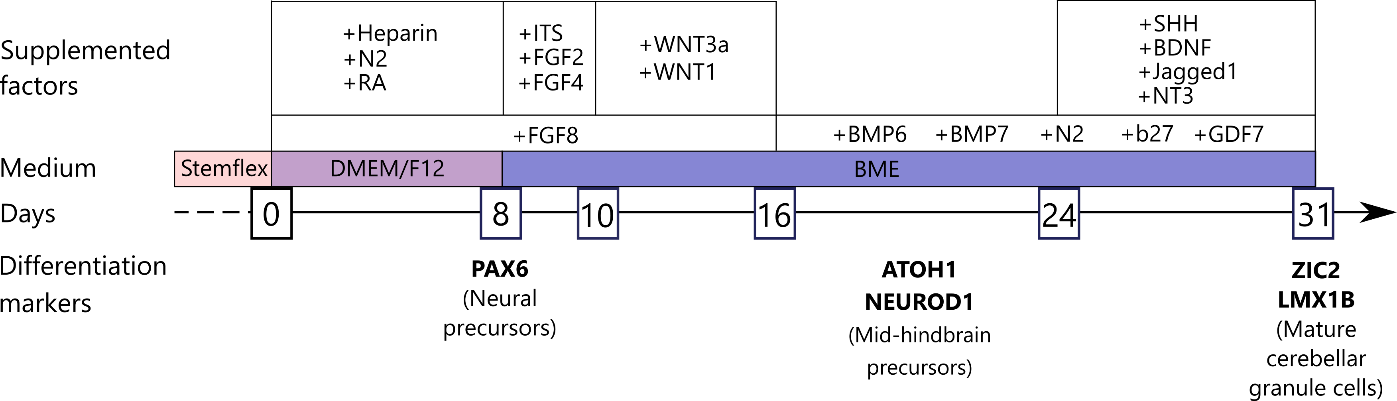


Online Resource Fig.9. Differentiation protocol over 31 days.


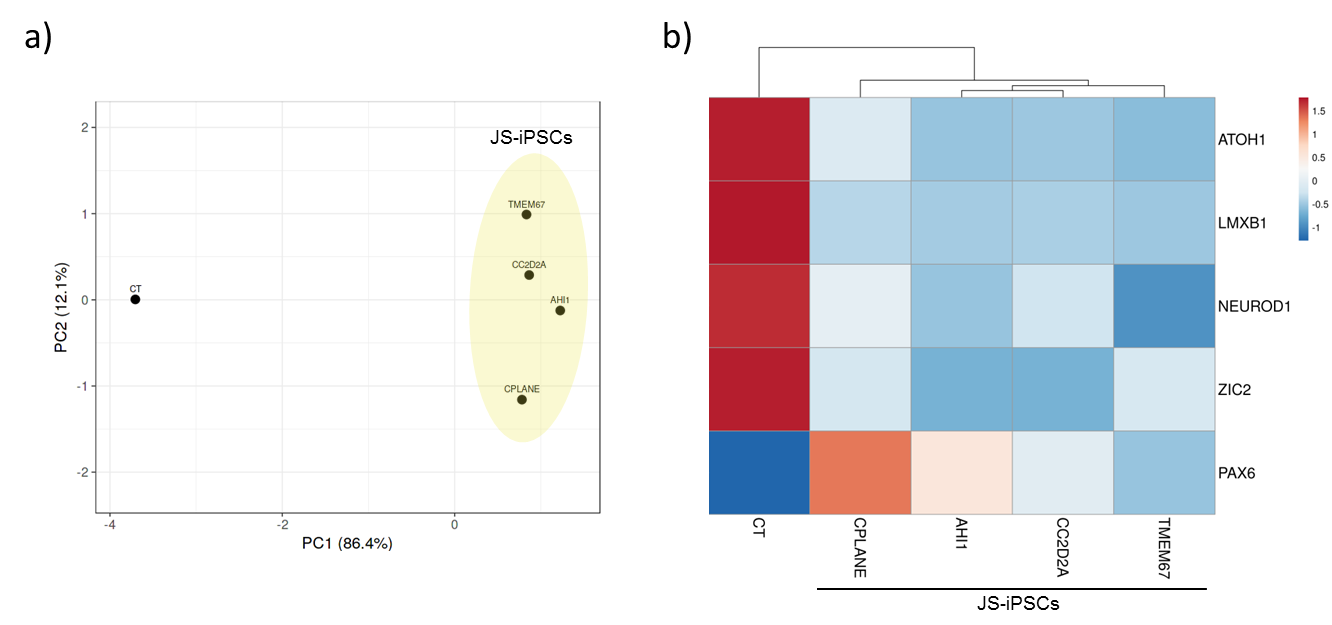


**Online Resource Fig.10.** Clustering of multivariate data analysis of the gene expression profile for markers shown in Figure 1 (*ATOH1*, *NEUROD1*, *ZIC2*, *PAX6*, *LMXB1*) at D24 of differentiation showing JS-iPSC lines clustered away from CT-iPSCs in PCA (a) and heatmap (b) analysis performed using ClustVis [1].


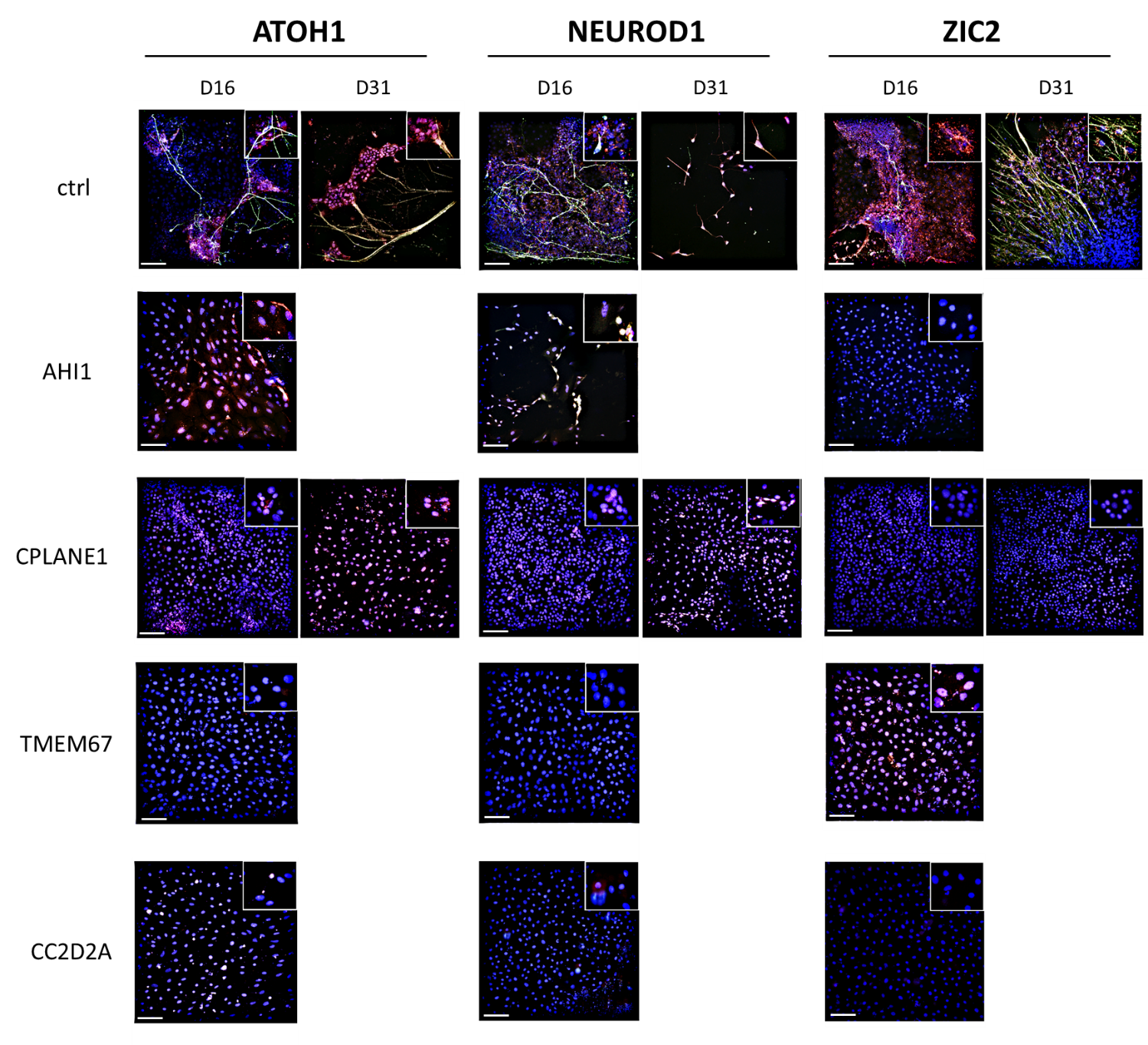


**Online Resource Fig.11.** Differentiation of iPSC lines towards mid-hindbrain and cerebellar lineages. Imunofluorescence staining for β3-TUBULIN (green) and common granule cells markers (red) ATOH1 (**a**), NEUROD1 (**b**) and ZIC2 (**c**) in CT-iPSCs (top panel) and JS-iPSC lines (lower panels) after 16 and 31 days of differentiation. Nuclei were counterstained with Hoechst (blue). Scale bar: 100 μm.


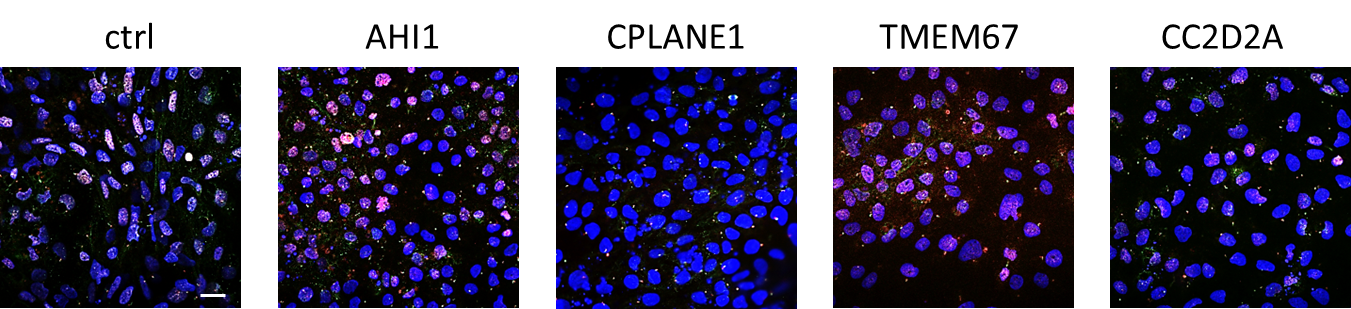


**Online Resource Fig.12.** Lower magnification immunofluorescence images showing cilia in iPSCs at day 8 of differentiation labelled with anti-AcTUBULIN (green), anti-PERICENTRIN (red) and Hoechst nuclear counterstain (blue). Scale bar: 20 μm.

**Online Resource Table 1.** qRT-PCR values for each differentiation marker. Relative fold change normalized values at D0 for each cell line, n=3.

|  | **Sample** | **D0** | **D8** | **D16** | **D24** | **D31** |
| --- | --- | --- | --- | --- | --- | --- |
| ***ATOH1*** | **ctrl** | 1 | 3.41 | 44.06 | 54.99 | 19.24 |
|  | ***AHI1*** | 1 | 1.17 | 2.83 | 1.53 | - |
|  | ***CPLANE1*** | 1 | 0.41 | 4.33 | 11.42 | 19.92 |
|  | ***TMEM67*** | 1 | 0.64 | 0.26 | 0.19 | - |
|  | ***CC2D2A*** | 1 | 0.59 | 0.12 | 2.20 | - |
| ***NEUROD1*** | **ctrl** | 1 | 4.98 | 15.31 | 17.51 | 4.66 |
|  | ***AHI1*** | 1 | 3.40 | 3.09 | 3.10* | - |
|  | ***CPLANE1*** | 1 | 0.51 | 1.38 | 6.71 | 9.69 |
|  | ***TMEM67*** | 1 | 0.70 | 0.81 | 0.54 | - |
|  | ***CC2D2A*** | 1 | 1.04 | 0.50 | 4.93 | - |
| ***ZIC2*** | **ctrl** | 1 | 1.29 | 1.27 | 2.02 | 1.13 |
|  | ***AHI1*** | 0 | 0 | 0 | 0 | - |
|  | ***CPLANE1*** | 0.5 | 0.04 | 0.17 | 0.41 | 0.05 |
|  | ***TMEM67*** | 0.33 | 0.71 | 0.65 | 0.43 | - |
|  | ***CC2D2A*** | 0 | 0 | 0 | 0 | - |
| ***PAX6*** | **ctrl** | 1 | 11.66 | 1.32 | 0.36 | 0.19 |
|  | ***AHI1*** | 1 | 4.36 | 1.73 | 16.43 | - |
|  | ***CPLANE1*** | 1 | 8.15 | 5.17 | 23.49 | 51.63 |
|  | ***TMEM67*** | 1 | 1.36 | 1.59 | 6.95 | - |
|  | ***CC2D2A*** | 1 | 10.60 | 21.61 | 11.21 | - |
| ***LMX1B*** | **ctrl** | 1 | 4.08 | 19.64 | 685.58 | 1131.28 |
|  | ***AHI1*** | 1 | 12.27 | 3.99 | 7.99 | - |
|  | ***CPLANE1*** | 1 | 8.91 | 6.56 | 32.03 | 53.92 |
|  | ***TMEM67*** | 1 | 0.92 | 0.49 | 0.43 | - |
|  | ***CC2D2A*** | 1 | 1.23 | 0.69 | 18.64 | - |

**Online Resource Table 2.** Primers used for qRT-PCR analysis of gene expression.

| **Gene** | **Forward primer** | **Reverse primer** |
| --- | --- | --- |
| *ATOH1* | GTCCGAGCTCGTACAAACG | GTGGTGGTGGTCGCTTTT |
| *NEUROD1* | CCTTCCTTTGATGGACCCC | GATTGATCCGTGGCTTTGG |
| *ZIC2* | CGAAAGAGAGAATGGGTCAAAGA | GCACATGCAGAGACCTCACTTC |
| *LMX1B* | TGTGCAAGGGTGACTACGAGAA | CCCATCTTCATCCTCGCTCTT |
| *PAX6* | GTGTCCAACGGATGTGTGAG | CTAGCCAGGTTGCGAAGAAC |
| *β-ACTIN* | AGAGCTACGAGCTGCCTGAC | AGCACTGTGTTGGCGTACAG |

**Supplementary Methods:**

*Stemness marker analysis:*

Immunofluorescence was carried out as described in 2.3 using antibodies against stemness markers OCT4 (Abcam Cat. No. AB19857) and TRA-1-60 (Abcam Cat. No. AB16288). qRT-PCR analysis of stemness gene expression was carried out as described in 2.4 using the following primers:

**Online Resource Table 3**. Primers used for qRT-PCR analysis of stemness gene expression.

| **Gene** | **Forward primer** | **Reverse primer** |
| --- | --- | --- |
| *OCT4* | TGTACTCCTCGGTCCCTTTC | TCCAGGTTTTCTTTCCTAGC |
| *NANOG* | CAGTCTGGACACTGGCTGAA | CTCGCTGATTAGGCTCCAAC |
| *SOX2* | GCTAGTCTCCAAGCGACGAA | GCAAGAAGCCTCTCCTTGAA |
| *ACTB* | ACCATGGATGATGATATCGC | TCATTGTAGAAGGTGTGGTG |

Karyotype analysis:

iPSC karyotype was checked either by molecular karyotyping or by chromosomal spread. Molecular karyotyping of iPSCs (passages 12 to 17) was performed at Life&Brain GmbH (Bonn, Germany) using the HumanOmni2.5 Exome-8 DNA Analysis BeadChip on genomic DNA extracted from each sample using the kit Zymo Research Direct-Zol RNA MiniPrep Plus (Euroclone Cat. No. R2072) [2]. For chromosome spreads, iPSCs were treated as described in [3] and at least 20 metaphases from each line were analysed following the International System for Human Cytogenomic Nomenclature (ISCN 2016) [3].

*Pluripotency assay:*

Trilineage differentiation was performed as described in [4] and analysed using the following primers:

**Online Resource Table 4**. Primers used for qRT-PCR analysis of germ layers gene expression.

| **Gene** | **Forward** | **Reverse** |
| --- | --- | --- |
| *PAX6* | GTCCATCTTTGCTTGGGAAA | TAGCCAGGTTGCGAAGAACT |
| *SOX1* | CACAACTCGGAGATCAGCAA | GGTACTTGTAATCCGGGTGC |
| *GATA4* | GGAAGCCCAAGAACCTGAAT | GTTGCTGGAGTTGCTGGAA |
| *FOXA2* | GTCCGACTGGAGCAGCTACTAT | CACGTACGACGACATGTTCA |
| *ACTA2* | GTCCCAGACATCAGGGAGTAA | TCGGATACTTCAGCGTCAGGA |
| *TBXT* | TGTACTCCTTCCTGCTGGACTT | AGCTTGTTGGTGAGCTTGACTT |

**References**

1. Metsalu T, Vilo J (2015) ClustVis: a web tool for visualizing clustering of multivariate data using principal component analysis and heatmap. Nucleic Acids Res 43:W566-570. https://doi:10.1093/nar/gkv468.
2. Mencke P, Boussaad I, Önal G, Kievit AJA, Boon AJW, Mandemakers W, Bonifati V, Krüger R (2022) Generation and characterization of a genetic Parkinson’s disease-patient derived iPSC line DJ-1-DelP (LCSBi008-A). Stem Cell Res 62:102792. https://doi:10.1016/j.scr.2022.102792.
3. Ali E, Ferraro RM, Guglielmi A, Lanzi G, Masneri S, Piovani G, Mazzoldi EL, Pollara L, Valente EM, Accorsi P, et al. (2021) Establishment of three Joubert Syndrome-derived induced pluripotent stem cell (iPSC) lines harbouring compound heterozygous mutations in CC2D2A Gene. Stem Cell Res 54:102430. https://doi:10.1016/j.scr.2021.102430.
4. Serpieri V, Orsi A, Mazzotta C, Cavan S, Rossi E, Scelsa B, Valente EM (2023) Generation of an iPSC line from skin fibroblasts of a patient with Joubert Syndrome carrying the homozygous loss of function variant c.787dupC in the AHI1 gene. Stem Cell Res 66:103002. https://doi:10.1016/j.scr.2022.103002.
